# From By-Products to Promising Bifunctional Food Ingredients: Physicochemical Characterization and Antioxidant and Emulsifying Improvement Evaluation Based on the Synergy of Phenolic Acids, Flavonoids and Tannins with Bovine Liver Hydrolysates

**DOI:** 10.3390/foods14132225

**Published:** 2025-06-24

**Authors:** Yufeng Duan, Xue Yang, Ruheng Shen, Li Zhang, Xiaotong Ma, Yuling Qu, Long He, Lin Tong, Guangxing Han

**Affiliations:** 1 College of Food Science and Engineering, Gansu Agricultural University, Lanzhou 730070, China; 2 Inner Mongolia Horqin Cattle Industry Co., Tongliao 028000, China; 3 Shandong Lvyrun Food Co., Ltd., Linyi 276300, China

**Keywords:** dual properties, microstructure, binding laws, emulsions

## Abstract

In recent years, bifunctional ingredients extracted and utilized from waste by-products as raw materials have received significant attention in the food production process. Previous studies have found that bovine livers possess both antioxidant and emulsifying potential; therefore, enhancing these dual properties is a current research focus. In this study, three different types of polyphenols (epigallocatechin gallate [EGCG], gallic acid [GA] and tannin [TA]) provide a reference on how to achieve better complexation of polyphenols with bovine liver hydrolysates (BLHs). Based on the molecular weight results, it was shown that the bovine liver peptides bind to polyphenols to form complexes with higher molecular weights. Furthermore, the binding affinities among the three complexes were as follows: TA > EGCG > GA. The emulsions stabilized by the coupling compounds contained more homogeneous and dense droplets (optical microscopy). Both the antioxidant properties and the emulsifying activity of all complexes were superior to those of bovine liver hydrolysates (BLHs) (*p* < 0.05), confirming synergistic effects that either flavonoids, phenolic acids or tannins possess with bovine liver hydrolysates. This combination provides an effective strategy for developing novel foods with specific functions.

## 1. Introduction

Proteins, as an important component of the food system, have always been favored for their unique interfacial properties and functional attributes. The liver, as one of the main organs for processing and utilizing by-products, has been demonstrated to have excellent nutritional quality and protein application potential, which is an important way to achieve effective utilization and high-value application of resources [1]. In addition, the liver undergoes hydrolysis to produce shorter peptides with lower molecular weights, which show better functional properties than natural proteins in terms of cellular regulation, immunomodulation, and antihypertensive, antimicrobial and metal chelating activities [2], but this process harms their interfacial properties [3], and exhibits instability [4]. Therefore, based on the previously identified good antioxidant potential of bovine liver hydrolysates and their emulsification capacity being constrained by enzymatic hydrolysis, we focused our research on how to improve their emulsification capacity, and to further enhance their antioxidant function, and the introduction of polyphenols provided an entry point for the development of dual-performance components.

Polyphenols are widely found in plant tissues, and not only do they possess good functional properties [5], but their abundant phenolic hydroxyl groups can also form complexes with proteins/peptides in food, thus altering their functional properties [6]. Zhang et al. [7] focused mainly on the alteration of the emulsification properties of different polyphenols with wheat germ, whey proteins and globulins; Zhou et al. [8] also chose identified tea polyphenols to be explored for bioavailability with cow’s milk proteins. These results suggest that the current study is slightly lacking in both the representativeness of the polyphenol choices and the plurality of functional studies. And most studies still focus on individual polyphenols (e.g., tea polyphenols) or flavonoids (catechins, quercetin, etc.) to improve the properties of hydrolysates or proteins. And flavonoids and their common C6-C3-C6 backbone are only one type of polyphenol and do not fully represent the peptide modification effects of all polyphenols, and there is a lack of representative phenolics to serve as a control group to verify that polyphenol modification patterns of bovine liver hydrolysates are consistent. Therefore, from a new perspective, it is worth exploring whether representative phenolic acids (benzene ring–carboxyl–hydroxyl) and tannins (sugar–phenolic acid) can be similar to flavonoids (C6-C3-C6 backbone) or better enhance the dual functionality of bovine liver hydrolysates. Additionally, it is important to investigate whether there is a consistent modification pattern for peptides among different types of polyphenols (phenolic acids, flavonoids and tannins). These issues still need to be investigated.

Structurally, the number and position of phenolic hydroxyl groups vary greatly, which affects their emulsifying activity and antioxidant properties. Among them, EGCG is among the most active flavanols found in tea polyphenols, and includes a phenylene glycol ring attached to a tetrahydropyran, an o-benzenetriol ring and a gallic acyl group (Figure 2B). The bioactivity of EGCG, and in particular its antioxidant activity, is dependent on the number and position of the hydroxyl groups on the ring, which determine their ability to interact with other components through hydrogen bonding or electron and hydrogen transfer processes [9]. Gallic acid (GA) belongs to a typical class of phenolic acids, and has high bioactivity due to its multi-hydroxyl structure. Because GA has four hydroxyl groups (Figure 2C), it is often added to protein systems as a biologically active substance to enhance the bioactivity of the protein system and improve the physicochemical properties of the protein system [10]. Tannic acid (TA), a hydrolyzed tannin composed of gallic acid esters of glucose (Figure 2D), interacts well with functional proteins such as sodium caseinate, gelatin and pea proteins due to its rich content of catechols and o-benzenetriol [11], and previous studies have shown that TA positively affects the properties of proteins or protein-based emulsions, such as by improving protein foam stability, emulsion physical stability and resistance to lipid oxidation [12]. Thus, tannins have potential applications in the design of protein-based food products.

**Figure 1:**
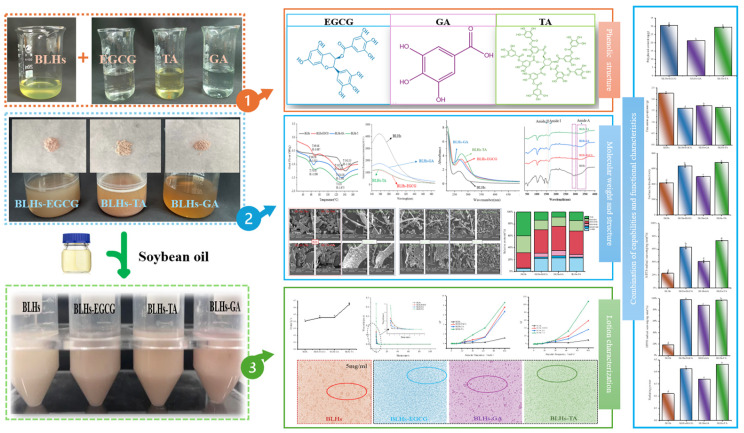
Experimental design process chart (Notes: 1. Solutions of bovine liver hydrolysate and three polyphenols; 2. Preparation of the solutions of three complexes and their freeze-drying sample; 3. Preparation of three complex solutions into crude emulsions; Lowercase letters represent significant differences among samples).

Based on the fact that EGCG, TA and GA contain different o-phenyltriphenol units, in addition to being typical features of flavanols, phenolic acids and tannins, the present experiments were carried out with complexes such as BLHs-GA/EGCG/TA. The entire experimental design process is shown in Figure 1, aiming to address the following questions: (1) to verify whether polyphenols can be an effective intervention strategy to enhance emulsification properties and antioxidant properties; (2) to explore whether certain consistent patterns are presented in the combination of different polyphenols and hydrolysates (that is, this study helps to analyze the rules of effects of polyphenols on the interface and function of hydrolysates); (3) to offer a reference for the structural design of food products and the exploration and utilization of novel bifunctional food ingredients, and to extend the applications of protein hydrolysates and polyphenols and their complexes in the fields of food and other industries.

## 2. Materials and Methods

### 2.1. Materials and Reagents

Bovine liver (Lanzhou, China), epigallocatechin gallate (purity ≥ 90%), gallic acid (purity ≥ 98.5%), and tannic acid (purity ≥ 96%) were used. Three analytical-grade polyphenols were purchased from Shanghai Yuan Ye Co., Ltd. (Shanghai, China).

### 2.2. Preparation of Hydrolysates

The preparation procedure here fully references the previous study [13], where bovine liver hydrolysates were prepared using ultrasound (500 W, 20 min)-assisted alkaline protease (where the amount of enzyme added is 0.4% of the substrate), freeze-dried, and then awaited analysis. BLHs were used as the abbreviation of sample name.

### 2.3. Preparation of Complexes

Minor modifications were made to previous methods [14]. BLHs (1 g) were dissolved in 49 mL of phosphate-buffered saline, and 0.1 g of phenolics was weighed and dissolved in 50 mL of PBS separately for 2 h before mixing with bovine liver hydrolysate. A total of 0.2% sodium propionate, 1 mL of 10 mol/L H_2_O_2_ and 0.25 g of ascorbic acid were added to the mixture, which was stirred uniformly using a magnetic stirrer for 24 h at room temperature (around 22–25 °C) and placed under refrigerated conditions (0–4 °C) for dialysis for 48 h. The entire experimental design is shown in Figure 1.

### 2.4. Characterization of Binding Capacity

#### 2.4.1. Polyphenol Binding Equivalents

The methodology for polyphenol binding equivalents [15] was slightly modified. Briefly, 1 mL of BLHs–polyphenol complex (1 mg/mL) was mixed with Folin–Ciocalteu reagent (0.25 mol/L, 0.5 mL) and protected from light for 5 min. Next, 1 mL of 15% sodium carbonate (Na_2_CO_3_, *w*/*v*) was added, and the samples were heated for 60 min (40 °C) and then cooled for 20 min.

#### 2.4.2. Determination of Free Amino Groups

Determination was conducted according to the referenced method [16].

### 2.5. Determination of Molecular Weight Distribution

This step was conducted with minor modifications [17], using an Agilent 1260 series high-performance liquid chromatography instrument (Agilent Technologies, Santa Clara, CA, USA). The detector is Agilent RID G1362A (Waters Ultrahydrogel, 300 × 7.8 mm, 500-250-120A, Milford, MA, USA). Temperature: 40 °C; mobile phase: 0.1 mol/L NaNO_3_; flow rate: 1 mL/min.

### 2.6. Characterization of Structures

#### 2.6.1. Secondary Structure Qualitative Analysis

A Fourier transform infrared spectrophotometer was used to measure the FTIR spectra of BLH, BLHs-EGCG, BLHs-GA and BLHs-TA complexes (Vetex70, Bruker, Bremen, Germany). The spectrum of the sample ranges from 4000 to 400 cm^−1^.

#### 2.6.2. Tertiary Structure Characterization

Fluorescence spectroscopy was tested using a F-7100 fluorescence spectrophotometer (Hitachi High-Technologies Corporation, Tokyo, Japan) according to the method of [18] with slight modifications. The excitation wavelength was 290 nm, and the emission spectrum was recorded at 300~500 nm. The slit width at both excitation and emission is 5 nm.

#### 2.6.3. Ultraviolet (UV) Absorption Spectroscopy

According to the method described by [19], ultraviolet spectroscopy scans were performed on the dissolved bovine liver hydrolysate and three complexes under the condition of a specific wavelength of 220–500 nm. UV-Vis absorption spectra were acquired using a Shimadzu UV-2600 spectrophotometer (Shimadzu, Kyoto, Japan).

#### 2.6.4. Determination of Surface Hydrophobicity

The surface hydrophobicity of the samples was determined using 1-aniline-8-naphthalenesulfonate as a fluorescent probe. A total of 20 μL of ANS solution (8 mmol/L) was added to 2 mL sample solution, and fluorescence intensity was determined with the same apparatus as described in Section 2.6.2. The standard curve of concentration and fluorescence intensity was established, and the slope was used to measure hydrophobicity.

### 2.7. Thermal Stability Characterization

The thermal stability of the four samples was determined by DSC (Differential Scanning Calorimetry), and the curves were recorded during the warming process. The temperature profile range was set to 20–180 °C, with a heating rate of 10 °C/min.

### 2.8. Scanning Electron Microscopy Micro-Characterization

The four samples, after freeze-drying, were analyzed for morphological features using an SEM at 1000× and 2000×.

### 2.9. Antioxidant Capacity Characterization

#### 2.9.1. Determination of Free Radical Scavenging Capacity

Referring to previous studies [20], 0.5 mL of the sample was mixed with DPPH ethanol solution (0.1 mmol/L, 3.5 mL) for 30 min. The absorbance value at this point was recorded as A_1_, then the DPPH solution was replaced by ethanol (95%) and the absorbance was recorded as A_2_. The sample was replaced by ethanol as a blank control, A_0_.

(1) $$DPPH\;scavenging\;activity\left(\%\right)=(1-\frac{A_{1}-A_{2}}{A_{0}})\times 100\%\;\;$$

Prepare 7 mM of ABTS solution and 2.45 mM of potassium persulfate solution, mixed in 1:1 volume and protected from light for 16 h [21]. Dilute with phosphate buffer (0.1 M, pH 7.4) until the absorbance value is 0.75. Add 2 mL ABTS^+^ solution to 2 mL sample. Measure the absorbance of sample solutions with different treatment times at 734 nm after 70 min. In the formula, A1 is the absorbance obtained by the sample with ABTS solution, and A0 is the absorbance obtained by PBS with ABTS solution.

(2) $$ABTS\;scavenging\;activity\left(\%\right)=(1-\frac{A_{1}}{A_{0}})\times 100\%\;\;$$

#### 2.9.2. Iron Reducing Power

The reducing powers of BLHs were determined by the method of Zhang et al. [22].

### 2.10. Determination of Emulsifying Capacity

#### 2.10.1. Preparation of Emulsions

Four samples, BLHS and BLHs-EGCG\GA\TA, were configured with an aqueous phase of 5 mg/mL, and soybean oil was added at a ratio of 9:1 between the aqueous phase and the oil phase, and a high-speed disperser was used to work at 15,000 rpm for 3–5 min, and to make the emulsions more homogeneous and well-mixed, ultrasonication was used for 10 min at a power of 150 W. A crude emulsion of the four samples was obtained.

#### 2.10.2. Emulsifying Activity

A total of 4.98 mL of 0.1% mass fraction of the configured SDS solution was added to 20 uL of each of the four crude emulsions, and the absorbance value was measured at 500 nm as A_0_.

(3) $$EAI\left(m^{2}/g\right)=4.66\times A_{0}$$

#### 2.10.3. Rheological Behavior

A disposable rubber-head dropper was used to absorb a suitable number of emulsion samples placed on a flat plate, and the excess samples around it were wiped off, the distance between parallel plates was set to 1 mm, and a dropper applied low-density silicone oil around the flat plate sample. The shear rate is 0.1 s^−1^~100 s^−1^, and the flow curve between the shear rate and the viscosity of the emulsion was recorded.

The variation in the energy storage modulus and loss modulus with angular frequency in the range of 0.1–100 rad/s was determined for an oscillatory strain of 0.1%.

#### 2.10.4. Optical Microscopy

A microscope was used to observe the distribution of emulsion droplets. A total of 6 μL of emulsion was placed on a slide and the coverslip was covered slowly to prevent foaming, and the distribution of the emulsions prepared from the four samples was observed at the same magnification [23].

### 2.11. Statistical Analysis

Significance analysis in this study was carried out using IBM SPSS Statistics 26 for a one-way ANOVA test and polynomial and Duncan’s analysis to obtain the mean, significant difference (*p* < 0.05), etc., where the data were marked with lowercase letters to show the results of significant differences between the data, and graphing was processed using Origin 2021 software.

## 3. Results and Discussion

### 3.1. Complex Binding Capacity

#### 3.1.1. Polyphenol Binding Capacity

**Figure 2:**
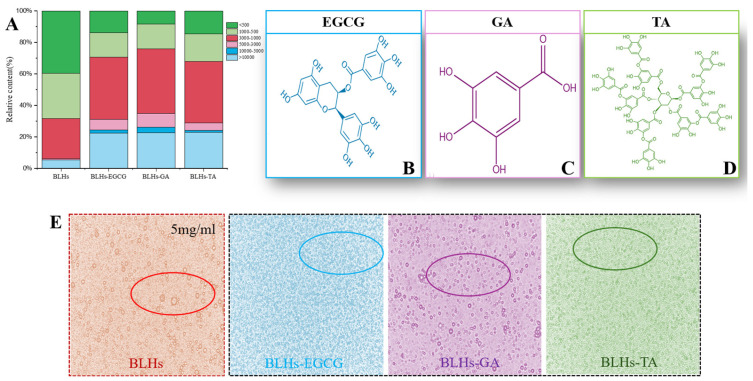
(**A**). Molecular weight distribution of bovine liver hydrolysate and its three complexes. (**B**). Schematic diagram of the molecular structure of EGCG. (**C**). Schematic diagram of the molecular structure of GA. (**D**). Schematic diagram of the molecular structure of TA. (**E**). Optical microstructures of the bovine liver hydrolysate emulsion and its three complex emulsions.

The number and location of hydroxyl groups have a significant effect on the activity of protein–polyphenol reactions [16]. The three structures of EGCG, GA and TA are shown schematically in Figure 2B–D. In terms of molecular structure, TA contains the largest number of phenolic hydroxyl groups, and the molecule has a certain degree of flexibility and can be regarded as a kind of multi-site crosslink, while the EGCG, with eight phenolic hydroxyl groups, can be used as a crosslinking agent, but its crosslinking effect is weaker than that of TA. The GA benzene ring molecule structure has three adjacent phenolic hydroxyl groups, which have a spatial resistance to prevent the bovine liver hydrolysates from combining with it. Therefore, their binding capacities were estimated as BLHs-TA, BLHs-EGCG and BLHs-GA, in descending order.

However, in terms of free radical reactivity, spatial site resistance and diffusion ability, the neighboring diphenol structure (catechol moiety) of EGCG is theoretically more reductive and can be easily oxidized to form quinone intermediates, which can form stable covalent bonds with nucleophilic groups (e.g., amino groups) of the polypeptide chain. Although TA contains more hydroxyl groups, its higher molecular weight may lead to its low diffusion efficiency in solutions. GA has a smaller molecule with high diffusion capacity but has a limited number of hydroxyl groups and lacks a neighboring diphenol structure to form sufficient interactions; thus, using EGCG and TA is relatively more advantageous in binding to bovine liver hydrolysates. To further validate their binding capacities, BLHs-EGCG, BLHs-GA and BLHs-TA complexes were prepared in the present experiments. In Figure 4F, Polyphenol Binding Capacity of BLHs-EGCG, BLHs-GA and BLHs-TA are, in descending order, those of BLHs-TA, BLHs-EGCG, and BLHs-GA. In connection with the above three polyphenols with different structures, it can be once again confirmed that the number of hydroxyl groups is the dominant factor in the binding of bovine liver hydrolysates.

#### 3.1.2. Free Amino Groups

Figure 4E compares the effects of the addition of three polyphenols, GA, EGCG and TA, on the free amino content of bovine liver hydrolysate. Compared with the bovine liver hydrolysate control, there was a significant difference in the free amino content of BLHs-GA, BLHs-EGCG and BLHs-TA (*p* < 0.05). Based on the results of the previous polyphenol binding equivalence assay, it was known that the binding capacities of the three polyphenols, GA, EGCG and TA, to bovine liver hydrolysates were, from the highest to the lowest, those of BLHs-TA, BLHs-EGCG and BLHs GA. In contrast, the free amino acid content was the opposite. Hydrogen bonds are usually formed by the hydroxyl oxygen of polyphenols and the amino group of proteins, so polyphenols with a stronger affinity for proteins will reduce the level of free amino groups in proteins [24].

### 3.2. Molecular Weight Distribution

Figure 2A shows the relative percentage of molecular weight of each component before and after the complexation of BLHs with EGCG, GA and TA. In the lower-molecular-weight fractions of 1000–500 and less than 500, the low molecular weights of the three complexes bound to the polyphenols were reduced, but still accounted for a large proportion, because the proteins were degraded to peptides after enzymatic degradation, and the low-molecular-weight substances of the peptides were still present even when bound to the polyphenols [25]. Among the higher-molecular-weight fractions greater than 10 kDa, BLHs accounted for 5.4%, while BLHs-EGCG, BLHs-GA and BLHs-TA maintained a share of about 22%, a 4.07-fold increase relative to BLHs. In addition, the same trend was observed in the 5–10 kDa, 3–5 kDa and 1–3 kDa fractions, which occurs because protein or peptide radicals preferentially attack polyphenols in the presence of large numbers of polyphenols. Covalent bonding directly attaches phenolic molecules to polypeptide chains, resulting in an increase in the molecular weight of the complexes, which is consistent with the findings of He et al. [26]. It is therefore reasonable to conclude that in the binding of polyphenols to proteins/peptides, the complexes tend to take the form of macromolecules.

### 3.3. Fourier Transform Infrared Spectroscopy

**Figure 3:**
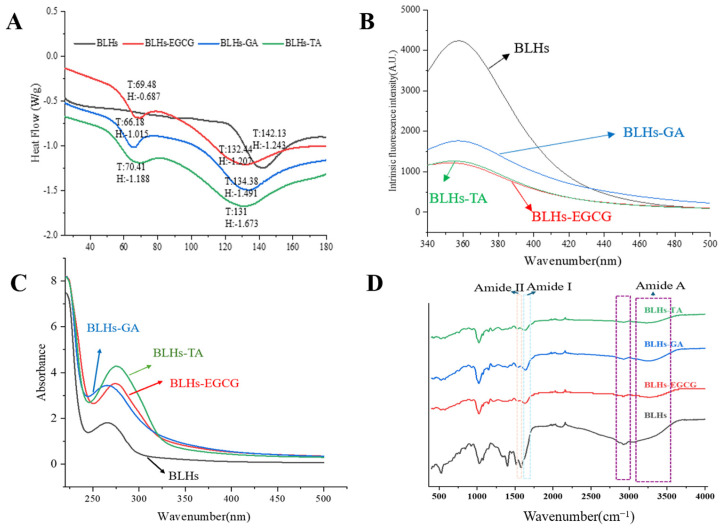
(**A**). DSC scanning result plot. (**B**). Fluorescence spectroscopy. (**C**). Ultraviolet spectroscopy. (**D**). Fourier infrared spectroscopy.

FTIR can reflect the presence of or change in functional groups or chemical bonds in molecules and qualitatively analyze samples [23]. Figure 3D shows the FT-IR spectra of the bovine liver hydrolysates containing the three polyphenols as well as the bovine liver hydrolysates themselves. Spectral and intensity changes were observed at 3100–3600 cm^−1^ (amide A), reflecting the stretching vibration of hydrogen bonding, with a redshift of the peak position in the amide A band. This may be due to the O-H stretching vibration induced by the incorporation of OH groups in the polyphenol molecule, which is related to the hydrogen bonding effect between polyphenols and proteins, suggesting a successful complexation of the polyphenol with the three polyphenols [27]. The peaks in the amide I band are in the range of 1700~1600 cm^−1^ and those in the amide II band are in the range of 1500~1600 cm^−1^ and are associated with C-O, C-N and N-H groups [28]. In this case, with the addition of polyphenols, the amide I band of the complex samples shifted towards higher wavenumbers, and this change suggests that the interaction of the bovine liver hydrolysates with the three polyphenols altered their secondary structure.

### 3.4. Fluorescence Spectroscopy

Aromatic amino acid residues in protein molecules, tryptophan, tyrosine and phenylalanine, can emit fluorescence under the excitation of a certain wavelength light source, and proteins containing these three amino acids are endogenous fluorescent substances, and tryptophan, tyrosine and phenylalanine residues show fluorescence peaks at 348 nm, 303 nm and 282 nm, respectively, because of the different chromophores. Therefore, fluorescence spectroscopy is often used to investigate the interactions between proteins and small molecules [29]. Since the fluorescence of phenylalanine residues in protein molecules is extremely weak and easily burst, the fluorescence is mainly generated by tyrosine and tryptophan. As shown in Figure 3B, the maximum emission wavelengths of bovine liver hydrolysates and their three complexes were in the range of 340–370 nm, with fluorescence peaks appearing at specific excitation wavelengths, so this study focused on the observation of changes in tryptophan fluorescence information. Comparative analysis of the change rule of endogenous fluorescence of bovine liver hydrolysates by three polyphenols, namely TA, EGCG and GA, revealed that the polyphenol molecules had a bursting effect and reduced the total fluorescence intensity. BLHs-TA, BLHs-EGCG and BLHs-GA showed an overall decreasing trend, which indicated that the addition of polyphenols interacted with the BLHs. Reflecting the changes in the fluorescent chromophore itself and its surrounding environment in protein molecules, the fluorescence bursting ability of the three polyphenols on BLHs was in the order of TA > EGCG > GA, and the amount of redshift in the fluorescence spectra of proteins depends on the binding capacity of polyphenols. Polyphenols can induce various changes in protein conformation through structure unfolding [14]. TA exposed tryptophan more than EGCG and GA, leading to the weakening of the fluorescence intensity of bovine liver hydrolysates and a weak redshift of the maximum emission wavelength, so we believe that TA has the strongest ability to change the tertiary structure of BLHs.

### 3.5. UV Spectrum

UV-Vis spectral changes can be used as evidence for structural changes in modified proteins [30]. Interaction of polyphenols with proteins induces redshifts and blueshifts in the absorption spectra of proteins, or enhancement and decolorization of signals [31]. Therefore, the UV-vis spectra of the couplings of EGCG, GA and TA were compared with BLHs. As shown in Figure 3C, there is a distinct absorption peak at wavelength 267 nm for all the bovine liver hydrolysates, which is mainly generated by aromatic amino acid residues such as tyrosine and tryptophan containing conjugated double bonds in the proteins [32,33]. The introduction of polyphenols into the bovine liver hydrolysate system resulted in an overall increase in UV absorption intensity. This finding agrees with earlier studies where similar results were seen when perilla seed meal protein was coupled with different polyphenols [34]. In this study the intensity of the UV absorption peak of bovine liver hydrolysates with TA at around 275 nm was significantly higher than those of bovine liver hydrolysates with EGCG at around 273 nm and GA at around 260 nm; this change in absorbance suggests that the addition of polyphenols alters the micro-environment around the protein chromophore and the extent of the increase varies with the polyphenol species. It is hypothesized that this phenomenon appears to be a result of the exposure of aromatic heterocyclic hydrophobic groups in the tryptophan and tyrosine residues of the molecule [35].

### 3.6. Surface Hydrophobicity

**Figure 4:**
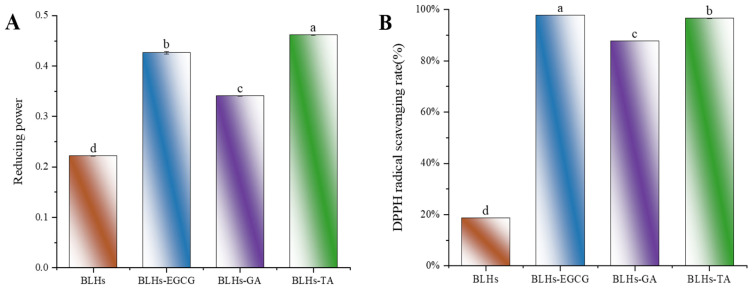

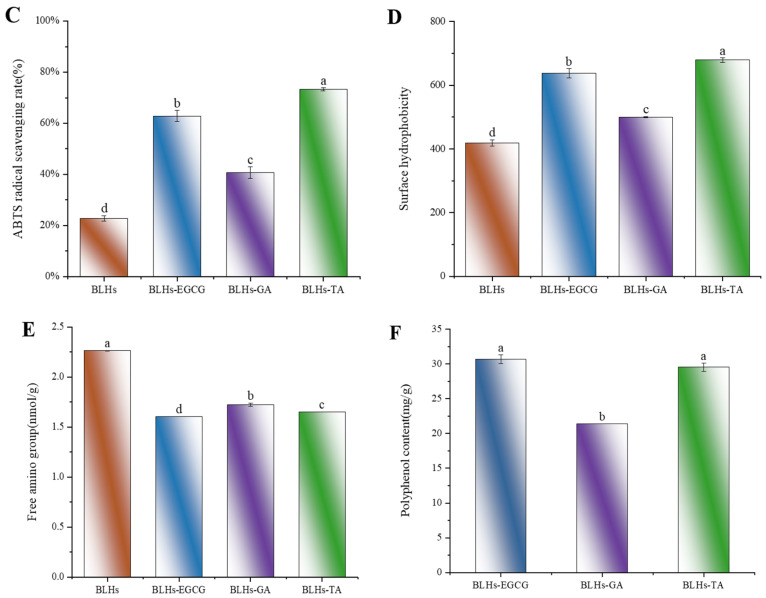
(**A**). Schematic representation of the reducing ability of BLHs, BLHs-EGCG, BLHs-GA and BLHs-TA. (**B**). DPPH radical scavenging of BLHs, BLHs-EGCG, BLHs-GA and BLHs-TA. (**C**). ABTS radical scavenging of BLHs, BLHs-EGCG, BLHs-GA and BLHs-TA. (**D**). Schematic representation of the changes in surface hydrophobicity of BLHs, BLHs-EGCG, BLHs-GA and BLHs-TA. (**E**). Schematic diagram of changes in free amino acid content. (**F**). Differences in polyphenol content of the three complexes (Note: Lowercase letters represent significant differences among samples).

Interfacial behavior of proteins is strongly influenced by surface hydrophobicity [36]. The changes in hydrophobicity of the binary complexes were further investigated using ANS as a fluorescent probe, in which the negative charge of the ANS sulfonic acid group binds strongly to the positive charge of the amino acid residues of peptides or proteins to form anionic pairs. The surface hydrophobicity of the bovine liver hydrolysates increased overall with the addition of different polyphenols. In similar studies, the interaction of whey protein and β-lactoglobulin with different phenolics and allyl isothiocyanate yielded the same observations [30]. Figure 4D shows the change in hydrophobicity of the surface after the addition of polyphenols. Each polyphenol led to an increase in H_0_, in the order GA < EGCG < TA (*p* < 0.05). The hydrophobicity of proteins is related to the number and configuration of protein polar groups. The increased hydrophobicity of the modified proteins can be explained by the structural differences of the different polyphenols. In addition, the modified complex proteins may undergo loosening. This loosening allows the ANS to reach some hydrophobic sites inside the molecule, which leads to an increase in the fluorescence intensity of the ANS in BLHs modified with the used phenols [30].

### 3.7. Differential Scanning Calorimetry Analysis

DSC is commonly recognized as an effective thermal analytical method for examining the interactions between proteins and phenolic compounds. The peak sizes are typically used as indicators of the thermal stability of the samples [34]. As shown in Figure 3A, the plots of BLHs modified by polyphenols are basically similar, all of which produce two peaks at 60–80 °C versus 140–160 °C, respectively, corresponding to the two peak melting temperatures (Tp), and the different peak temperatures indicate that the number of conjugates is both increasing and decreasing to different degrees [34]. The thermal absorption peaks of BLHs-EGCG, BLHs-GA and BLHs-TA were significantly lower compared with the denaturation temperatures of BLHs, suggesting that covalent binding induced by different structural polyphenolic compounds reduced the thermal stability of bovine liver hydrolysate. The thermal stability of proteins is related to the structural changes in the α-helix and the mobility of the branched chain during the modification process [37]. The addition of polyphenols may disrupt the original secondary structure of the bovine liver hydrolysate, making it more likely to unfold when heated and accelerating the denaturation process. Furthermore, based on the molecular weight results demonstrating that polyphenols and bovine liver hydrolysates form macromolecules as a result of binding, it is possible that these aggregates may exhibit a decrease in apparent thermal stability, assuming that phase separation occurs as a result of the increased hydrophobicity of these aggregates when subjected to heat.

### 3.8. Antioxidant Capacity

To measure the changes in antioxidant activity of EGCG-, GA- and TA-modified BLHs, we used three in vitro antioxidant evaluation methods, namely reducing power, ABTS and DPPH, which are widely used for the determination of antioxidant activity, and shown in Figure 4A–C. The reducing power value of BLHs was 0.222. After modification, there was a significant difference (*p* < 0.05) between the reducing powers of BLHs-EGCG, BLHs-GA and BLHs-TA, which increased approximately 1.5-2.1-fold when compared to the BLH control group. In the ABTS scavenging activity assay, the free radical scavenging capacity was consistent with the reduced capacity, and the antioxidant capacity was BLHs-TA > BLHs-EGCG > BLHs-GA in descending order, all of which were significantly greater than that of BLHs (*p* < 0.05). This difference in activity may be related to the increase in the number of hydroxyl (OH) groups grafted onto BLHs and when polyphenols bind to proteins, The free hydroxyl group on the benzene ring can act as an antioxidant, and the polyphenol–protein system can scavenge free radicals, form more stable reaction products and terminate the free radical chain reaction [38]. In addition, we found that BLHs modified with EGCG\TA recorded DPPH scavenging rates of (97.8% and 96.5%) at 1 mg/mL, respectively, possessing higher DPPH scavenging viability compared to GA-modified BLHS, and these results suggest that grafting of bovine liver hydrolysates with different kinds of polyphenol radicals can have the effect of enhancing antioxidant capacity.

### 3.9. Emulsifying Properties

**Figure 5:**
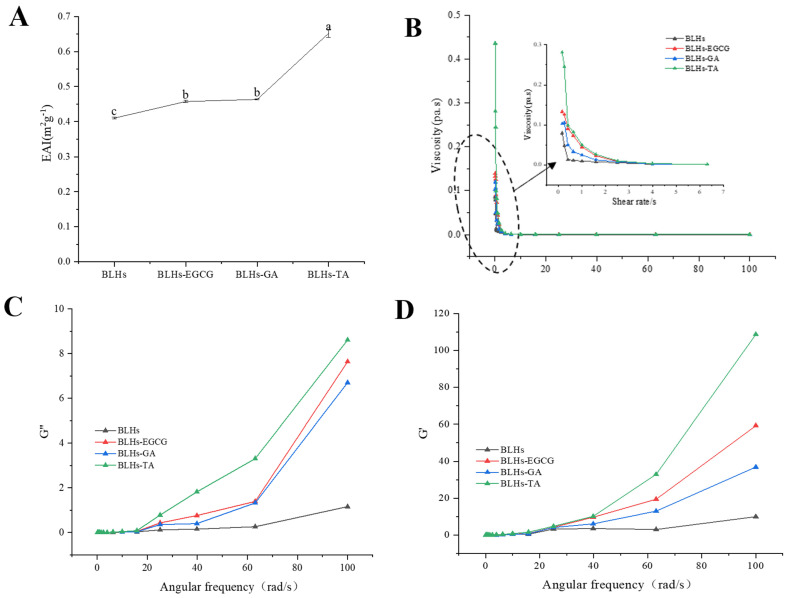
(**A**). Emulsifying activity of four crude emulsions. (**B**). Schematic of the variation in viscosity with shear rate for four emulsions. (**C**). Schematic of the variation in energy storage modulus with angular frequency. (**D**). Schematic diagram of loss modulus variation with angular frequency (Note: Lowercase letters represent significant differences among samples).

EAI is the ability of proteins or other emulsifiers to adsorb onto the surface of newly formed droplets, and this ability reduces the interfacial tension between the oil and water phases, thereby stabilizing the emulsion [34]. The EAIs of the three complexes are shown in Figure 5A. Compared with bovine liver hydrolysates, the EAI was significantly increased, and the emulsification activity index (EAI) of the mixed group showed BLHs-TA > BLHs-GA > BLHs-EGCG > BLHs, which indicated that the interaction of EGCG\TA\GA with bovine liver hydrolysates facilitated the increase in the EAI, which might be related to the exposure of aromatic amino acids of the bovine liver hydrolysates after their modification by polyphenols and the unfolding of the peptide chain [39]. The flexibility of peptide/protein hydrolysates may be enhanced when combined with polyphenols, and such complexes may be more effective in reducing the surface tension of emulsion droplets [40]. Some studies have shown that silk gum protein hydrolysates covalently coupled with quercetin or rutin have higher emulsifying activity [41]. Similar findings were found in the emulsion system stabilized by the proanthocyanidin–soybean meal hydrolysate coupling [19]. Thus, bovine liver hydrolysates in the form of polyphenol complexes possess some potential to enhance emulsification properties, while similar conclusions can be drawn under optical microscopy.

### 3.10. Rheological Properties

The viscosity profiles of fresh emulsions constructed from bovine liver hydrolysates after treatment were plotted as apparent viscosity versus shear rate (Figure 5B). The emulsion exhibited non-Newtonian pseudoplastic (shear thinning) behavior. The shear thinning behavior of dispersions could be explained by their high molecular weight [27]. In addition, when the shear rate sufficiently overcomes the Brownian motion, the droplets in the emulsion become more ordered along the flow field and offer less resistance to flow, thus exhibiting reduced viscosity [42]. The shear thinning characteristics of the cow liver hydrolysate covalently bonded to polyphenols were more pronounced than in the control. This result may be due to a thicker interfacial layer and a higher resistance to flow of the droplets [43].

To investigate the viscoelastic properties of the samples, small amplitude oscillation tests were carried out in this study. The storage modulus (G′) and loss modules (G″) are two viscoelastic parameters that express gel strength. As shown in Figure 5C,D, the G′ and G′ values gradually increased with increasing angular frequency, and the G′ values were significantly higher than the G″ values. The samples exhibited typical gel-like behavior, and the complex samples changed significantly to being better than the beef liver hydrolysate.

### 3.11. Optical Microscope

Optical microscopy images of the emulsion stabilized by bovine liver hydrolysates and its couplings are shown in Figure 2E. All samples showed spherical structures, indicating that the samples were absorbed at the oil–water interface to stabilize the droplets. However, larger spherical bubbles with aggregation were observed in the emulsions stabilized by BLHs only. Beef liver hydrolysate has a relatively weak adsorption capacity at the oil–water interface, and some instability of the emulsion was also observed in soybean meal and potato hydrolysate [19,44]. Emulsions stabilized by the coupling compounds contained significantly smaller droplets, suggesting that coupling of phenolic compounds to BLHs may promote droplet disruption or inhibit droplet aggregation [45]. Among them, BLHs-EGCG and BLHs-TA systems are uniformly distributed and show some similarity, and the emulsions are more stable, and BLHs-GA emulsions have a small amount of aggregation and present a relatively poor effect, but in the case of complexation with phenolic compounds, all three show improved emulsification properties of proteins under the microscope, and in addition, it has been shown that the combination of phenolics alters hydrophobicity and flexibility and increases their surface activity [37]; on the other hand, the combination of bovine liver hydrolysates with polyphenols may have increased the thickness and charge of the lipid droplets, enhancing their flocculation stability by increasing the spatial repulsion between the droplets [46].

### 3.12. Scanning Electron Microscopy

**Figure 6:**
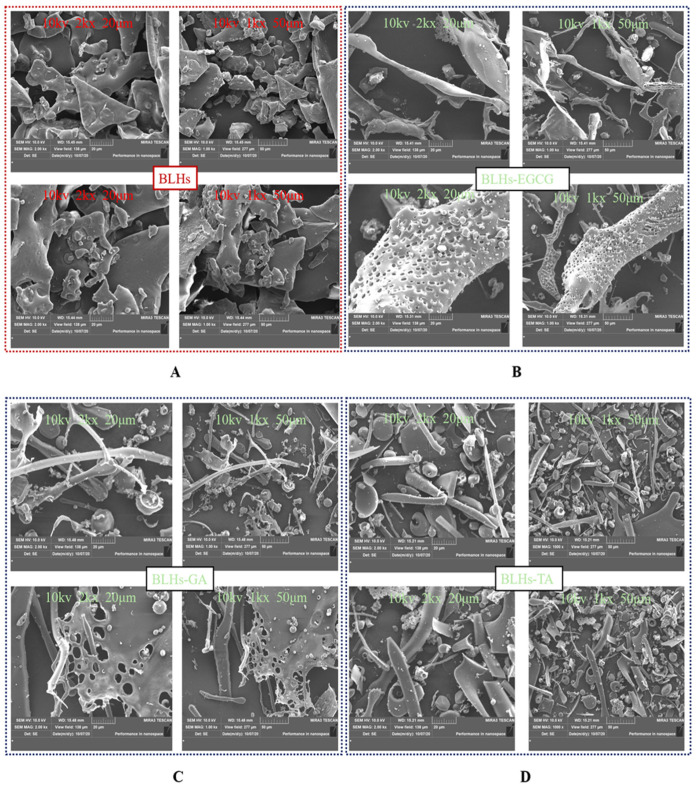
(**A**). Microstructure of BLHs at 2kx\1kx magnification. (**B**). Microstructure of BLHs-EGCG at 2kx\1kx magnification. (**C**). Microstructure of BLHs-GA at 2kx\1kx magnification. (**D**). Microstructure of BLHs-TA at 2kx\1kx magnification.

SEM images of BLHs and BLHs-EGCG, BLHs-GA and BLHs-TA are shown in Figure 6. In the control BLHs, it was observed that BLHs showed a lamellar shape with a roughened surface of the matrix, and relatively large volume, and many aggregates with thick walls were observed, whereas the structure of BLHs-EGCG, BLHs-GA and BLHs-TA was looser, which indicated that the structure of BLHs was damaged to some extent due to the interactions with the different polyphenols and the formation of new complexes [47]. BLHs-EGCG, BLHs-GA and BLHs-TA all have higher fragmentation rates than BLHs, and BLHs-EGCG exhibits an overall dendritic structure with small globular objects attached to it. BLHs-GA and BLHs-TA exhibit globular and rod-like structures, and similar structural disruptions were observed in the covalent binding of wheat gluten hydrolysates to chlorogenic acid, both of which together formed rod-like or globular structures. Among them, BLHs-TA is the most compact, while BLHs-GA is relatively sparse, which is related to the structural differences in polyphenols [26]. This is similar to the structural compactness observed by Zhao et al. when they studied the interaction of casein with tannic acid and gallic acid, respectively [45].

## 4. Conclusions

The results showed that the interaction system of EGCG, GA, TA and BLHs provided an effective intervention strategy to enhance the antioxidant and emulsifying properties of bovine liver hydrolysate. TA exhibited greater BLH binding capacity and antioxidant and emulsifying activities than EGCG or GA. In addition, with the addition of polyphenols, the complexes all showed some consistent patterns, such as the increase in molecular weight; the action of polyphenols with BLHs increased the site resistance and repulsive force between droplets, which reduced the interfacial tension at the oil–water interface, prevented the aggregation of droplets and significantly altered the interfacial rheological properties, etc. These findings provide a basis for the binding of hydrolysates and phenolics. However, there is still a lack of validation in terms of site of action and antioxidant application, emulsification stability, etc. Therefore, the next step is to focus on the experimental content and activity validation of the binding site to improve the experimental system and provide a reference for further extension of the application.

## Data Availability

The original contributions presented in the study are included in the article, further inquiries can be directed to the corresponding author.
